# Brain activation during the observation of real soccer game situations predicts creative goal scoring

**DOI:** 10.1093/scan/nsab035

**Published:** 2021-03-24

**Authors:** Christian Rominger, Karl Koschutnig, Daniel Memmert, Ilona Papousek, Corinna M Perchtold-Stefan, Mathias Benedek, Andreas R Schwerdtfeger, Andreas Fink

**Affiliations:** Department of Psychology, University of Graz, Graz 8010, Austria; Department of Psychology, University of Graz, Graz 8010, Austria; Institute of Exercise Training and Sport Informatics, German Sport University of Cologne, Cologne 50933, Germany; Department of Psychology, University of Graz, Graz 8010, Austria; Department of Psychology, University of Graz, Graz 8010, Austria; Department of Psychology, University of Graz, Graz 8010, Austria; Department of Psychology, University of Graz, Graz 8010, Austria; Department of Psychology, University of Graz, Graz 8010, Austria

**Keywords:** tactical creativity, sports decision, artificial intelligence

## Abstract

Creativity is an important source of success in soccer players. In order to be effective in soccer, unpredictable, sudden and at the same time creative (i.e. unique, original and effective) ideas are required in situations with high time pressure. Accordingly, creative task performance in soccer should be primarily driven by rapid and automatic cognitive processes. This study investigated if functional patterns of brain activation during the observation/encoding of real soccer game situations can predict creative soccer task performance. A machine learning approach (multivariate pattern recognition) was applied in a sample of 35 experienced male soccer players. The results revealed that brain activation during the observation of the soccer scenes significantly predicted creative soccer task performance, while brain activation during the subsequent ideation/elaboration period did not. The identified brain network included areas such as the angular gyrus, the supramarginal gyrus, the occipital cortex, parts of the cerebellum and (left) supplementary motor areas, which are important for semantic information processing, memory retrieval, integration of sensory information and motor control. This finding suggests that early and presumably automatized neurocognitive processes, such as (implicit) knowledge about motor movements, and the rapid integration of information from different sources are important for creative task performance in soccer.

## Introduction

Similar to creative ideas, successful solutions in soccer are often flexible, unique, original and surprising (see, e.g., Memmert, 2013). In fact, there is increasing evidence that, in addition to physical fitness, cognitive and especially creativity-related processes are key components in soccer (see, e.g., Vestberg *et al.*, 2012, 2017; Huijgen *et al.*, 2015; Memmert, 2017; Fink *et al.*, 2019; Rominger *et al.*, 2020a). In adhering to standard definitions of creativity, which conceptualize creativity as involving both novelty/originality and practicability/effectiveness (e.g. Runco and Jaeger, 2012), creative solutions in soccer are not only original/novel but also of a particular value (i.e. the target orientation is to score a goal). Soccer players need to focus their attention on specific conditions of the soccer scenario, to anticipate the behavior of other players, to think of possible passes and shots and to choose the most promising next move along with an effective motor execution for scoring a goal. Imagining creative moves also involves the search and retrieval of task-relevant information stored in memory (e.g. soccer-specific rules, technical knowledge about the execution of the pass or move and trained standard situations). Additionally, in order to generate a creative and effective move, soccer players need to evaluate the efficacy and appropriateness of the imagined move and inhibit inappropriate, potentially less successful solutions. Moreover, they are also continuously required to adapt to changing constraints and need to timely execute automatized (motor) procedures in response to predictive cues in opponents (e.g. Abernethy *et al.*, 2008; Wright *et al.*, 2013; Furley and Memmert, 2015; Aquino *et al.*, 2016; Roca *et al.*, 2018; Cardoso *et al.*, 2019).

In contrast to creative activities such as painting or poetry, where long-lasting ideation and elaboration processes might be associated with a higher creative performance outcome (e.g. Beaty and Silvia, 2012; Rominger *et al.*, 2018), in rapidly changing sport situations, cognitive processes operate under time pressure and require immediate responses (Vickers and Williams, 2017; Roca *et al.*, 2018). So far, however, most neuroscientific studies investigating creativity-related processes in sports have focused on neurocognitive processes during pre-defined ideation and elaboration periods (e.g. Fink *et al.*, 2019; Rominger *et al.*, 2020a). In these studies, participants were shown brief video clips of real soccer game situations. In a critical situation, the image was frozen (signaling the start of the idea generation period), and participants were asked to imagine themselves as the acting player of the attacking team and, depending on the experimental condition, to think of either a typical/conventional or a creative/original sequence of moves that might lead to a goal. During this idea generation period, brain activity was assessed by means of Electroencephalography (EEG) (Fink *et al.*, 2018; Rominger *et al.*, 2020a) or Functional magnetic resonance imaging (fMRI) (Fink *et al.*, 2019), and participants were not allowed to speak. Only during the subsequent response period, they were required to vocalize their ideas, which were then assessed with respect to originality/creativity according to a pre-defined evaluation scheme. The results revealed that differences in creative soccer task performance were associated with activity patterns in a mainly left-lateralized network of brain regions, primarily involving the cuneus, middle temporal gyrus and the Rolandic operculum, which are known to support the processing of multimodal inputs from different sensory, motor and perceptual sources (Fink *et al.*, 2019). In a quite similar vein, EEG findings suggested that creative soccer task performance was associated with brain activity in networks supporting visuospatial attention and movement imagery (Fink *et al.*, 2018; Rominger *et al.*, 2020a). Although this procedure is well suited to investigate brain activation associated with a pre-defined idea generation period following the observation of the soccer scenes, it may be even more realistic to consider the spontaneous and automatized processes occurring at the very moment of stimulus exposure, that is, during the game situation (i.e. during encoding these soccer scenes; see, e.g., Wimshurst *et al.*, 2016; Roca *et al.*, 2018; Cardoso *et al.*, 2019).

This study, therefore, focused on the brain activity associated with more spontaneous and automatized processes implicated during the observation and encoding of the soccer scenario. This is particularly motivated by recent behavioral findings showing that the allocation of attention to informative locations (Furley *et al.*, 2010; Roca *et al.*, 2018, 2020) and automatized (motor) processes (Cardoso *et al.*, 2019) are specifically important for successful soccer performance. For instance, in Roca *et al.* (2018), soccer players had to interact with a representative life-size video-based simulation of attacking soccer situations. An interesting finding of this study was that more creative as compared to less creative players employed a broader attentional focus, including more fixations of shorter duration and toward more informative locations of the display (as assessed by a portable eye-movement registration system).

In the present study, experienced soccer players were shown brief video clips of real soccer game situations while fMRI was assessed. Unlike former studies (Fink *et al.*, 2018, 2019; Rominger *et al.*, 2020a), participants were not asked to generate either a creative or conventional solution but rather to generate task solutions in a self-driven and thus in a more naturalistic manner. They were told that in the given soccer scenario, various solutions were possible and they should think of the most promising moves to score a goal. Brain activity was assessed during both observation/encoding of the soccer scenario and the subsequent ideation/elaboration phase. This procedure hence allows to determine whether creative soccer task performance can be predicted by brain activity already occurring during the observation and the immediate processing of the stimulus material. We applied pattern recognition analysis by means of machine learning, which is becoming increasingly popular in the field of cognitive neuroscience. In contrast to univariate methods, which consider brain features as independent from each other, the multivariate pattern recognition method is more sensitive in detecting spatially distributed effects and uses the joint information of all brain features, which might more precisely capture the organization of brain functions (Schrouff *et al.*, 2013, 2018). This approach yields important new information about the multivariate patterns of brain activity implicated with more automatized processes during the observation/encoding of the soccer scenario *vs* explicit thinking and elaborating on a solution during subsequent idea generation phases.

## Methods

### Participants

Thirty-five experienced male soccer players without a history of neurological or psychiatric disease participated in this study. The mean age was 26.00 years (s.d. = 4.99, min = 18, max = 36 years). All participants had been actively playing soccer for at least 10 years with an average of 19.71 years of active soccer playing experience (s.d. = 5.30, max = 29). They reported to have played soccer for *M* = 3.33 h per week (s.d. = 3.58). Three (8.57%) of the participants were active soccer coaches and three (8.57%) were soccer referees. The highest soccer league participants indicated they had ever played was the third highest national league (*n* = 8). Participants were ranked with respect to the highest soccer league they have ever been playing in their career as a measure of expertise. All participants had normal or corrected to normal vision and gave written informed consent to participate in this study. The study was approved by the authorized local ethics committee.

### Experimental task during fMRI assessment

**Fig. 1.**
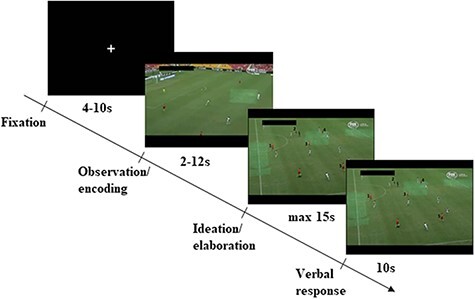
Schematic time course of the computerized soccer task.

Participants worked on a modified version of a soccer decision-making task (Fink *et al.*, 2018, 2019; Rominger *et al.*, 2020a). Each trial started with a jittered baseline period (fixation cross, 4–10 s) followed by the period of observation, where brief video clips of a real soccer game situation were presented (between 2- and 12-s length; see Figure 1). The stop of the soccer scene (encoding/observation period) marked the beginning of the explicit and pre-defined idea generation and elaboration period. The last scene of the video clip was frozen and players of the attacking team were labeled with a number. Participants were instructed to imagine themselves as the acting player of the attacking team and to generate a move that would most probably lead to a goal. Participants had to press a button as soon as they had thought of a creative solution/move (max 15 s). Then, during a fixed verbal response period of 10 s, they had to vocalize the imagined move briefly (e.g. pass to 3 and then shot). Participants were instructed to name only one solution per response interval. The oral responses were recorded and transcribed for further analyses. In total, 41 scenes were presented in randomized order.

### Creative task performance

To assess creative task performance, all responses (moves) were quantified with respect to their creativity. Creative task performance scores were based on the first move to score a goal, which had been pre-rated by four Union of European Football Associations (UEFA) A licensed soccer coaches. These experts viewed all possible first moves to score a goal along with the respective video and rated the creativity of each solution on a scale from 1 to 7. The UEFA A coaches were instructed to rate the solutions with respect to novelty/originality and usefulness/effectiveness (regarding goal scoring), in adhering to standard definitions of creativity which emphasize both originality and effectiveness (Runco and Jaeger, 2012; see, e.g., Silvia *et al.*, 2008 for a similar approach considering more than one dimension for creative performance ratings). Basically, the entire set of video clips allowed a broad range of creative task solutions. The inter-rater reliability was indexed by the Intraclass Correlation Coefficient (ICC) >0.80 (for more detailed information, see Fink *et al.*, 2018, 2019). Ratings were averaged across all scenarios to obtain a creative task performance score per participant.

### fMRI data acquisition

Whole-brain imaging was conducted on a Siemens Skyra 3T system with a 32-channel head coil. Functional images were acquired using a multiband (CMRR) sequence covering the whole brain (repetition time [TR] = 1400 ms, echo time [TE] = 30 ms, flip angle = 65, multiband factor = 4, 2.5 mm isotropic voxel size, 60 slices with no gap). The slice package was tilted approximately 30° relative to the AC-PC line. Structural images were acquired using a submillimeter T1-weighted magnetization prepared rapid acquisition gradient echo sequence (TR = 2200 ms, TE = 218 ms, flip angle 8, inversion time [TI] = 1000, 0.88 mm isotropic voxel size). Visual stimuli were presented using the software Presentation (Neurobehavioral System, Albany, CA, USA) and delivered via a 32″ Full HD monitor (NNL, NordicNeuroLab, Bergen, Norway) located at the back of the scanner bore. Verbal response was captured with an MR-compatible microphone system (FOMRI-III; Optoacoustics, Mazor, Israel). Button presses were collected using an MRI compatible fiber optic response pad (Current Designs Inc., Philadelphia, PA, USA).

### fMRI data pre-processing and general linear model analysis

The standard pre-processing pipeline fMRIprep version 1.1.7 (Esteban *et al.*, 2018, 2019), which is based on *Nipype* 1.1.3 (Gorgolewski *et al.*, 2011, 2018), was used for anatomical and functional pre-processing. Each T1-weighted image was corrected for intensity non-uniformity (INU) using ‘N4BiasFieldCorrection’ (Tustison *et al.*, 2010, ANTs 2.2.0), skull-stripped using ‘antsBrainExtraction.sh’ version 2.2.0 (using OASIS). Spatial normalization to the ICBM 152 Nonlinear Asymmetrical template version 2009c (Fonov *et al.*, 2009) was performed through non-linear registration with ‘antsRegistration’ (ANTs 2.2.0, Avants *et al.*, 2008). Brain tissue segmentation of cerebrospinal fluid (CSF), white matter (WM) and gray matter (GM) was estimated on the brain-extracted structural image using ‘fast’ (FSL 5.0.9, Zhang *et al.*, 2001).

A reference volume and its skull-stripped version were generated as a first step in the functional pre-processing. A deformation field to correct for susceptibility distortions was then estimated based on two echo-planar imaging references with opposing phase-encoding directions, using ‘3dQwarp’ (Cox and Hyde, 1997). Based on the estimated susceptibility distortion, an unwarped blood oxygenation-level dependent (BOLD) reference was calculated for a more accurate co-registration with the anatomical reference. The BOLD reference was then co-registered to the T1w reference using ‘flirt’ (FSL 5.0.9, Jenkinson *et al.*, 2002), with the boundary-based registration (Greve and Fischl, 2009) cost function. Co-registration was configured with nine degrees of freedom to account for distortions remaining in the BOLD reference. Head-motion parameters with respect to the BOLD reference (transformation matrices, and six corresponding rotation and translation parameters) are estimated before any spatiotemporal filtering using ‘mcflirt’ (FSL 5.0.9). Functional images were then slice-time corrected (‘3dTshift’) and resampled to MNI152NLin2009cAsym standard space. Several confounding time series were calculated: framewise displacement (FD, following the definitions by Power *et al.*, 2014) and three region-wise global signals (CSF, WM and within whole brain mask). Additionally, a set of physiological regressors were extracted to allow for component-based noise correction (Behzadi *et al.*, 2007). Principal components are estimated after high-pass filtering the pre-processed BOLD time series (using a discrete cosine filter with 128-s cutoff). For anatomical noise (aCompCor), six components were calculated within the intersection of the aforementioned mask and the union of CSF and WM masks calculated in T1w space, after their projection to the native space of each functional run (using the inverse BOLD-to-T1w transformation). Resampling was performed with a single interpolation step using ‘antsApplyTransforms’ (ANTs), configured with Lanczos’ (1964) interpolation to minimize the smoothing effects of other kernels.

First-level analysis was conducted using the general linear model (GLM) implemented in SPM12 (vers 7487, Wellcome Trust Centre for Neuroimaging). The following MRI model regressors were convolved with the canonical hemodynamic response function: observation/watching, ideation/elaboration, button press and verbal response (all with varying durations). This resulted in a varying number of available volumes per participant with *M* = 877 (min = 802, max = 974). Additionally, 16 regressors of no interest (CSF, WM, global signal, FD, 6 × aCompCor, 6 × Motion) were entered in the design matrix. The contrast images (subtracting baseline from activation) representing the patterns of brain activation in response to (i) the observation period and to (ii) the ideation/elaboration period served as an input for the multivariate pattern recognition analysis.

### fMRI data analyses

We analyzed the data in two stages. First, the multivariate pattern recognition analysis was conducted to assess if and which brain regions are informative for the prediction of creative task performance during encoding/observing and during ideation/elaboration, separately. Second, we subsequently conducted linear regression-based GLM analysis predicting creative task performance within the Region of interest (ROIs) of the significant prediction model. This allows to answer why the ROIs might be informative in the predictive model (for similar logic, see Schrouff *et al.*, 2018).

### Machine learning: multivariate pattern recognition analysis

Pattern recognition analysis (PRoNTo toolbox, Version 2.1; Schrouff *et al.*, 2013) rather than a mass-univariate approach was used to investigate the relationship between creative soccer task performance and the overall pattern of brain activity during (i) encoding/observing the soccer scenarios and (ii) ideation/elaboration. Specifically, we set up two feature sets based on a regression model. The first feature set A was designed to analyze the relationship between the creative performance outcome and brain activation evoked by watching the brief soccer scenes. The second feature set B was analogous to the feature set A, but here, we investigated the relationship between the brain activation during the pre-defined ideation/elaboration period and participants’ creative task performance. For both feature sets, we trained and tested a multi-kernel (MKL) regression model with the creative performance scores (Schrouff *et al.*, 2018). By means of this MKL approach, it is possible to simultaneously learn and combine different kernels based on atlas-based brain regions (automated anatomical labeling atlas, AAL-Atlas; Schrouff *et al.*, 2018). Each feature set was normalized and mean centered and cross-validation was performed on the basis of a Leave-One-Subject-Out scheme. In detail, each model trains and tests the contribution of each pre-defined anatomical region for the decision function and further trains and tests the contribution of each voxel within each region. The MKL algorithm optimizes the weights of each kernel, and this process takes place inside a cross-validation loop. After all iterations were calculated, the weights of all ROIs can be obtained. For each of the 116 atlas-based brain regions, a linear kernel was computed based on the activation of each voxel within the respective region. Finally, to evaluate the significance of the prediction accuracy statistics, a permutation test was conducted (10 000 iterations; see, e.g., Portugal *et al.*, 2019; Schrouff *et al.*, 2018 for 1000 iterations). It was counted how many times the absolute value of the *r* (or mean squared error [MSE]) metric with the permuted labels (i.e. creative task performance) was equal to or higher than the one obtained with the correct labels, which were used for the multivariate pattern recognition (e.g. *p *= [number of permutations, where *r*_permuted_ ≥ *r*_correct labels_]/10 000; for a detailed overview of this approach, see Schrouff *et al.*, 2018; Portugal *et al.*, 2019).

### Additional GLM statistics indicating the direction of the association between creativity and brain activation within the ROIs

Since weight maps should not be interpreted as statistical parametric maps (Portugal *et al.*, 2019), we conducted a linear regression based on the GLM implemented in SPM12 within the ROIs to determine if the identified brain regions predicted creative task performance positively or negatively. Since this approach should only give us information about the direction of activation, we applied a very liberal threshold of *P* < 0.005 (uncorrected) within the ROIs. This supplemental procedure is analogous to Schrouff *et al.* (2018), who suggested univariate follow-up tests for significant brain regions involved in the model (ROIs) in order to provide additional information for interpretation.

## Results

### Pattern recognition analysis

**Fig. 2.**
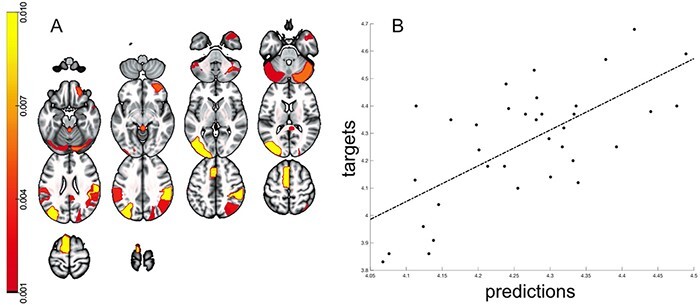
(A) ROI weights of brain areas involved in the prediction model of creative task performance in soccer. (B) The image illustrates the prediction of creativity by means of the multivariate pattern recognition analysis (*r* = 0.68, *P* < 0.01).

As illustrated in Table 1, feature set A (i.e. period of the observation of the real soccer scene) explained 46.24% of the variance in creative soccer task performance (*P* < 0.01). In contrast, feature set B (i.e. ideation/elaboration period) was not significant and explained <0.10% of soccer task performance (Figure 2A). These results indicate that brain activity during encoding/watching the soccer scene but not during the subsequent ideation/elaboration phase significantly predicted creative soccer task performance.

**Table 1.** Two prediction models for creative task performance in soccer

	*r*	*P*	MSE	*P*
Feature set A: encoding/observing	0.68	0.0028	0.02	0.0027
Feature set B: ideation/elaboration	−0.03	0.4949	0.07	0.4660

Although several cortical and subcortical regions contributed to the prediction model, the highest ROI weights and therefore the most discriminative voxels were found for the left middle occipital gyrus (MOG), the supramarginal gyrus (SMG), the angular gyrus (AG), the supplementary motor area (SMA) and various regions of the cerebellum (Table 2, Figure 2), as well as the right middle orbitofrontal cortex (OFC).

**Table 2.** Brain regions included in the significant prediction model A, which was based on brain activation during the observation of real soccer scenes

Rank	Brain regions	ROI weight (%)	ROI size (vox)
1	Occipital_Mid_L	17.59	3186
2	SupraMarginal_R	16.8	1598
3	Supp_Motor_Area_L	15.16	2034
4	Cerebelum_Crus1_R	8.49	2026
5	Vermis_3	8.06	230
6	Frontal_Mid_Orb_R	7.8	732
7	SupraMarginal_L	5.56	1206
8	Angular_R	4.35	1558
9	Cingulum_Post_R	3.51	323
10	Cerebelum_Crus1_L	2.64	2239

The weight of the region indicates their contribution to the model.

### Additional GLM statistics to indicate brain activation increases or decreases during the observation of real soccer scenes

**Fig. 3.**
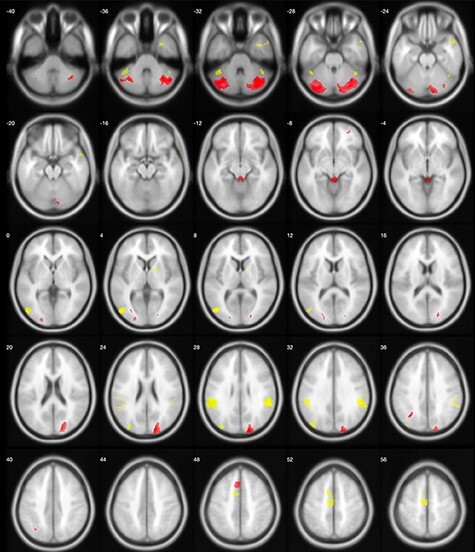
GLM analyses separately for the ROIs as identified by the pattern recognition analysis. Positive predictions are indicated by yellow color, and negative predictions are indicated by red color. Since for this analysis, only the direction of the activation is of interest, a very liberal threshold was applied (*P* < 0.005, uncorrected).

As illustrated in Figure 3, activation in the left MOG, the left and right SMG, the right AG, as well as specific parts of the left SMA and the cerebellum positively predicted creative performance. However, the right superior occipital, large parts of the cerebellum, parts of the left SMA and the right middle OFC showed negative associations with task performance.

### Behavioral results

There was a significant correlation of soccer-specific expertise (i.e. highest soccer league) with soccer task performance (*r *= 0.49, *P* = 0.003), which indicates better performance in soccer players with higher expertise. However, neither training hours per week (*r *= 0.25, *P* = 0.148) nor the years participants have been actively playing soccer (*r *= 0.18, *P* = 0.292) were significantly associated with creative task performance.

## Discussion

This study showed that a specific brain activation pattern during the observation and encoding of real soccer scenes significantly predicted the creativity of task solutions. In contrast, brain activity during an explicit ideation/elaboration period, which directly followed this watching period, did not predict creative soccer task performance. This finding suggests that the basis for creative task performance is laid already during a very early stage of the game situation, that is, during the viewing and encoding of the soccer scenario.

The identified brain network that significantly predicted creative soccer task performance involved brain regions which have been found to be important for creativity in other domains as well. This especially applies to the AG as well as the SMG (see, e.g., Fink *et al.*, 2009; Benedek *et al.*, 2018; for overviews, see Boccia *et al.*, 2015; Pidgeon *et al.*, 2016). These areas are associated with semantic processes, the integration of information and the retrieval of knowledge (Binder *et al.*, 2009). The positive association between creative task performance and the activation in the left SMG might indicate declarative and knowledge-based processes during watching the soccer scenes that subsequently facilitate creative soccer moves. This is nicely in line with a recent investigation of Roca *et al.* (2020), who assessed cognitive thought processes of professional soccer players in soccer game situations. Roca *et al.* reported more statements of planning and evaluation in more creative soccer players tasked to shoot a goal under time pressure. Similarly, the right SMG and the right AG were also positively associated with task performance in the present study, which might indicate greater involvement of associative functions as well as visual-spatial attention (Seghier, 2013). Specifically, the allocation of (visual) attention seems to be of high importance in sports (Furley *et al.*, 2010; Hüttermann and Memmert, 2017). Roca *et al.* (2018) reported that more creative soccer players showed a broader attentional focus in sports situations (e.g. more fixations of shorter duration to more informative locations; Vickers and Williams, 2017; Roca *et al.*, 2020). The involvement of the visual cortex in the soccer observation period in the current predictive model further corroborates this notion, most likely indicating effective visual processing of relevant stimuli. In addition, the activation of the left MOG is in line with creativity studies in other domains (e.g. Ellamil *et al.*, 2012; Aziz-Zadeh *et al.*, 2013; Boccia *et al.*, 2015), which is interpreted as mental imagery of possible solutions to a given open problem (Boccia *et al.*, 2015; Pidgeon *et al.*, 2016).

The left SMA was also part of the prediction model and is responsible for motor planning of the right extremities (Kaas and Stepniewska, 2002). This might signal the imagination of (motor) movements during the observation of the brief soccer scenarios (La Fougère *et al.*, 2010) and is in accordance with studies, reporting activation of motor-related brain areas during (i) creative task performance in soccer (Fink *et al.*, 2018, 2019; Rominger *et al.*, 2020a), (ii) creative ideation (predominantly) in the visual domain (Ellamil *et al.*, 2012; Boccia *et al.*, 2015; Saggar *et al.*, 2015, 2017; Rominger *et al.*, 2018; for overview, see Pidgeon *et al.*, 2016; Chen *et al.*, 2020) and (iii) the identification of soccer moves, action anticipation and mirroring of movements in sport (Wright *et al.*, 2013; Wimshurst *et al.*, 2016). The inclusion of the vermis and the cerebellum in the prediction model further indicates movement mirroring and imagination during the encoding/observation period. These areas provide adequate, smooth and quick execution of trained, implicit and automatic motor actions and are important for embodied problem-solving (Wright *et al.*, 2013; Koziol *et al.*, 2014). Taken together, the involvement of motor-related brain areas is well in line with studies indicating that the players’ action repertoire is important for creative performance in soccer (Caso and van der Kamp, 2020; see also Memmert and Roth, 2007) and strengthens the assumption that creative soccer performance may be defined as a players’ disposition to show movements outside the box (Santos *et al.*, 2018).

Finally, the absence of most frontal areas in the significant prediction model is an important finding, since the activation of frontal areas (e.g. inferior frontal gyrus and dorsolateral prefrontal cortex) was reported for creative ideation in many domains (Gonen-Yaacovi *et al.*, 2013; Boccia *et al.*, 2015; Chen *et al.*, 2020). In the present study, only the middle OFC of the right hemisphere seemed to be involved in creative task performance. However, right OFC activation during the observation period was negatively correlated with creative performance, possibly indicating that creative soccer players do not need to immediately evaluate the appropriateness of their ideas during game play (Huang *et al.*, 2018) and therefore exhibited less cognitive effort during the observation of real soccer game situations (for similar findings in the context of procedural tactical knowledge, see Cardoso *et al.*, 2019). This is in line with the assumption that highly creative soccer performance relies more strongly on fast, automatized and spontaneous processes than on frontal executive control processes (Costa *et al.*, 2018).

This study also provides important implications for creativity research in general and might not be restricted to creative task performance in sports. Although processes involved in the achievement of creative task performance in soccer seem to already take place during the exposure to the soccer scenes, the identified brain areas included in the prediction model are known in other domains of neuroscientific creativity research as well (Boccia *et al.*, 2015; Pidgeon *et al.*, 2016; Chen *et al.*, 2020). In addition, this study adds evidence to neuroscientific creativity studies investigating the time course of creative thinking, which suggest that earlier stages of creative ideation may require less executive control and more associative and automatized modes of thinking (Agnoli *et al.*, 2020; Schwab *et al.*, 2014; Beaty *et al.*, 2015; Rominger *et al.*, 2018, 2019, 2020b; Zhou *et al.*, 2018). The strong impact of the early stages of the creative thinking process on the ideation outcome might be one reason why Stevens and Zabelina (2020) reported a good discrimination between more and less creative brain states at a very early time window by means of machine learning of EEG data. The current findings are in accordance with these observations and underline the value of automatic and spontaneous modes of thinking for creative cognition (Mednick, 1962; Benedek and Jauk, 2018). Nevertheless, former neuroscientific studies also indicated the importance of an elaboration period for the creative ideation performance (Fink *et al.*, 2018, 2019). This divergence in findings might be well explained by the assumption that long-lasting ideation processes are more important when explicit ‘be creative’ instructions are applied and fast and automatized processes are favored when spontaneous thoughts (without explicit instructions) are required.

As a potential limitation of this study, it is important to note that the predictive model identified in this study needs replication, since the performance of pattern recognition depends on the sample size and the effect size (Jollans *et al.*, 2019). Furthermore, the regression model constitutes a mainly descriptive neuroscientific approach, with the objective to generalize from training and to learn some properties of the data (Schrouff *et al.*, 2013). Nevertheless, machine learning has the potential to discover new features that are not expected a priori. Despite these limitations of the reported weight maps, we were able to support our initial hypothesis that brain activation during observing/encoding of ongoing soccer scenes is suited to predict creative performance in the soccer task. It is important to note that the non-significant prediction model of brain activity during the ideation/elaboration period might not be observed due to undersampling in more creative people, since response times (and available volumes) were not associated with task performance. Additionally, motion should not be a problem for the present findings, since the experimental paradigm has the important advantage that motion almost solely occurs during the pre-defined verbalization phase (see Figure 1), which was not part of the pattern recognition analysis. Furthermore, motion was unrelated to creative task performance. Finally, following Forthmann *et al.* (2017) who used separate measures of uncommonness, remoteness and cleverness to assess overall creativity, future studies may assess creative task performance in soccer by aggregating the ratings of originality and effectiveness. However, in line with the present study, Silvia *et al.* (2008) delivered evidence for good reliability and validity of a creativity rating, where judges were instructed to consider several dimensions when making their ratings.

Taken together, the present findings indicate for the first time that the brain activation of experienced soccer players during viewing a soccer game situation significantly predicted the creative quality of their moves toward scoring a goal. This strengthens the assumption that creative task performance in soccer may strongly depend on automatized and spontaneous modes of thinking, operating at a very early stage of stimulus processing.

## Data Availability

The data that support the findings of this study are available at https://openneuro.org/datasets/ds002878.
